# Prevalence of Osteopathologies in Children and Adolescents After Diagnosis of Acute Lymphoblastic Leukemia

**DOI:** 10.3389/fped.2020.00509

**Published:** 2020-08-26

**Authors:** Michael M. Schündeln, Pia K. Hauffa, Martin Munteanu, Cordula Kiewert, Nicole Unger, Jens J. Bauer, Berthold P. Hauffa, Corinna Grasemann

**Affiliations:** ^1^Pediatric Hematology and Oncology, Department of Pediatrics III, University Hospital Essen, University Duisburg-Essen, Essen, Germany; ^2^Pediatric Endocrinology and Diabetology, Department of Pediatrics II, University Hospital Essen, University Duisburg-Essen, Essen, Germany; ^3^Department of Endocrinology, Diabetes and Metabolism, University Hospital Essen, University Duisburg-Essen, Essen, Germany; ^4^Division of Rare Diseases, Department of Pediatrics, St. Josef-Hospital, Ruhr-University Bochum, Bochum, Germany

**Keywords:** acute lymphoblastic leukemia, bone health, childhood malignancies, osteopathologies, survivorship, vitamin D

## Abstract

**Background:** Impaired bone health is a late effect of childhood malignancies which can be difficult to detect in juvenile survivors. It may, however, lead to compromised quality of life, or even permanent disability later in life due to osteoporosis, pain or fractures if left untreated. Acute lymphoblastic leukemia (ALL) is the most frequent childhood malignancy with an over 85% five-year survival. ALL and its treatment cause bone alterations in adults, but little information on the bone health status in juvenile survivors is available.

**Objective:** To report data on skeletal late effects in juvenile survivors of childhood ALL based on a comprehensive assessment of bone health and to assess the influence of a vitamin D deficiency on bone health in this cohort.

**Methods:** In a single center cross sectional study 128 pediatric patients (11.9 ± 4.76 years) with a mean follow up of 5.88 ± 3.75 years after diagnosis of ALL were recruited. The bone health status of the survivors was assessed based on clinical examination, review of medical records, biochemical and radiographic analyses, by clinical experts. A score which utilized 8 different parameters was formed and used to assess the effect of a vitamin D deficiency on bone health.

**Results:** In this cohort, 18% of survivors displayed overt osteopathologies as defined by clinical expert assessment. Impaired bone health, defined by at least one pathological screening parameter, was detected in 77%. Despite recommendations for adequate vitamin D supplementation, 15% displayed a vitamin D deficiency associated with hyperparathyroidism. The applied score identified survivors with osteopathologies with high sensitivity and specificity. The median score did not differ between patients without and with severe vitamin D deficiency.

**Conclusion:** Our findings suggest that impaired bone health and osteopathologies are common skeletal late effects following treatment of childhood ALL. Major contributing factors are BMT, irradiation and older age at diagnosis. Vitamin D deficiency likely accounts for hyperparathyroidism in some patients but does not seem to further affect bone health in this cohort. Survivors of ALL need thorough surveillance to investigate bone health, since bone morbidity is common and still poorly understood. Early detection and appropriate intervention may improve bone health.

## Introduction

Over the past decades, long-term survival rates in childhood malignancies and especially in acute lymphoblastic leukemia (ALL) have increased substantially with improved and adapted treatment regimens (1–3). In the growing group of survivors, late effects from cancer and its treatment are an increasing burden compromising health and quality of life. Endocrine late effects in general play a major role in survivorship morbidity (4–7). Bone health has been shown to be impaired in a significant number of children and adults surviving pediatric malignancies (8). Mostoufi-Moab and Halton review the subject for patients with childhood ALL (9). At time of diagnosis there is an incidence of vertebral fractures of up to 16% in the pediatric age group (10) and 72% develop osteonecroses, which often are asymptomatic (11). Patients undergoing treatment for ALL are particularly affected by low bone mineral content and low bone mineral density (BMD) (12, 13). Some studies report this to persist until adulthood (14, 15) while others report a normalization over time, especially in patients without cranial irradiation (16).

Beyond low bone mineral content and fractures many other factors may account for impaired bone health in patients with ALL, such as alterations in bone metabolism, chronic bone pain and stunted growth (17–20). In addition, the hormone vitamin D plays an important role in the maintenance of calcium homeostasis and thereby bone health in childhood and adolescence (21). Given its additional functions such as regulation immunity and cellular differentiation, an optimal vitamin D status may be important especially for patients with cancer (22). Therefore, the assessment of bone health in a population at risk for bone disease should account for all of the above. This is a difficult and complex task, in particular since the assessment of bone metabolism depends on age- and pubertal stage-appropriate interpretation of biochemical surrogate parameters.

In this manuscript, we describe skeletal late effects and vitamin D status in a cohort of juvenile survivors of childhood ALL based on a comprehensive assessment of bone health.

## Methods

### Patients

All patients who had received chemotherapy for acute lymphoblastic leukemia in the past and were either undergoing maintenance therapy or follow-up at the oncology outpatient clinic from August 2012 until August 2014 were invited to participate in this single-center cross-sectional study. Patients were recruited year-round to balance seasonal influences.

Data regarding ALL stratification and treatment of the patients is presented in Table 1. 134 patients were eligible to participate. Of those, 6 declined for personal reasons. Of the recruited 128 patients, 69 were male, and 59 were female. The patients had the following diagnoses: B-lineage ALL (*n* = 107), T-lineage ALL (*n* = 13), Philadelphia chromosome-positive ALL (*n* = 3), biphenotypic ALL (*n* = 2), mature B-lineage ALL (*n* = 3). The median initial blast count at diagnosis was around 4,000/μl with a wide range between 0 and 600,000/μl. All patients were treated as per the respective ALL-BFM protocols (23). Accordingly, 26 patients underwent allogeneic bone marrow transplantation (BMT). 11 patients underwent cranial irradiation only, 17 total body irradiation only and 5 patients received both treatments.

**Table 1:** Stratification and treatment of the cohort.

	**Fraction of total**	**Percentage**
Gender (male/female)	69/59 (128)	54/46
Diagnosis	B lineage: 107; T-lineage: 13; Ph positive: 3; biphenotypic: 2; mature B-lineage: 3	83.6/10.2/2.3/1.6/2.3
Risk-group (nonHR/HR)	92/36 (128)	71.9/28.1
CNS status (1/2/3)	97/21/7 (125)	77.6/16.8/5.6
Bone marrow transplant (BMT)	26/128	20.3
Relapse	19/128 (19 patients; 20 relapses)	14.8
Graft-vs.-host disease (GvHD)	18/128	14.1
Total body irradiation only	17/128	13.3
Cranial irradiation only	11/128	8.6
Total body irradiation and cranial irradiation	5/128	3.9

*Total numbers or fractions are displayed as well as the percentage for gender, diagnosis, risk-group [Non-high risk (nonHR) vs. high risk (HR)], central nervous system involvement (CNS Status; 1, negative; 2, low level leukemic blasts in spinal tab; 3, CNS disease), bone marrow transplant (BMT), relapse, graft-vs.-host disease (GvHD) of all grades, total body irradiation and cranial irradiation*.

### Clinical Parameters and Questionnaire

Clinical and anamnestic parameters were assessed as previously described in detail (24). Briefly, a physical examination, including determination of weight, height, and pubertal status was performed. Patients were also asked to complete a standardized questionnaire regarding vitamin D, calcium and nutritional supplement intake, screen hours and hours of physical activity per day (24). In addition, the patients were asked for presence of bone pain in the form of regularly occurring (on more than half of the days in the last month), spontaneous, back pain or exercise related knee pain. Pubertal development was assessed by a pediatric endocrinologist according to Tanner staging. Standard deviation scores (SDS) for pubertal development were calculated using “Puberty Plot Web Application” by Stef van Buuren (http://vps.stefvanbuuren.nl/puberty/) [accessed, May 12, 2020, (25)]. The program is based on a Dutch population study (26) and calculates pubertal SDS for breast development, pubic hair stage and testicular volume. While the calculation is based on the Tanner stages for breast development and pubic hair stage, the application allows only discrete measures for testicular volume (i.e., 2, 3, 4, 8, 12, 16, 20, and 25 ml). Therefore, volume measurements that fell in between these values, which were determined using an orchidometer, were rounded to the closest possible measurement (e.g., a measurement of 6 ml was rounded to 8 ml for this study). Skeletal age was determined by an experienced pediatric radiologist according to the method of Greulich and Pyle using X-ray images of the left hand (27).

### Chart Review

The following parameters were obtained from the patients charts: The subtype of ALL including the CNS status, the risk stratification according to the individual patient's protocol (standard/medium/high risk), the cumulative dosage of chemotherapy as well as type and dosage of irradiation dosage patient “as treated” and the steroid dosage as prednisone equivalent dosage (28). In patients who underwent a BMT the occurrence of graft vs. host disease (GvHD) and its grade, length, and treatment was documented.

Upon chart review, fractures which had occurred after the diagnosis of ALL were recorded. Vertebral fractures or more than two subsequent fractures of long bones were categorized as “pathological fractures.”

### Laboratory Tests

The following biochemical parameters of growth, pubertal status, bone turnover, and vitamin D metabolism were assessed in serum or plasma samples as part of the routine diagnostic laboratory workup in the central laboratory of the University Hospital Essen: 25-OH vitamin D (ng/ml); 1,25-(OH)_2_ vitamin D (pg/ml); serum phosphate (mmol/l), serum calcium (mmol/l), albumin (g/dl), total serum alkaline phosphatase, TSAP (U/l); bone-specific alkaline phosphatase, BAP (U/l); insulin-like growth factor-1, IGF-1 (ng/ml), PTH (pg/ml), and Osteocalcin (ng/ml).

Additionally, the urinary calcium to creatinine ratio (mg/mg) as well as markers of bone resorption including N-terminal telopeptide, NTX (nmol bone collagen equivalent (BCE)/mmol creatinine) and deoxypyridinoline, DPD (mg/g creatinine) were assessed in spot urine samples. Pediatric reference ranges were available and applied for all parameters. Serum IGF-1 levels were expressed as SDS values, according to age and sex, based on the data from Blum and Breier (29). To calculate the IGF-1 SDS, we used the software tool “SDSEasy,” (Mediagnost, Reutlingen, Germany).

### Bone Densitometry

BMD was examined via dual-energy X-ray absorptiometry (DXA) (Lunar Prodigy, GE-Healthcare, Madison, WI, USA) in a subgroup of patients. BMD was assessed at the lumbar spine (L1–L4; anteroposterior view) and the left femoral neck. Z-scores (DXA Z) were calculated for the lumbar spine measurements based on age specific normal values (30, 31). A single investigator blinded to the clinical status of the patients was responsible for all BMD measurements. Height-adjusted Z-scores (HAZ) from DXA were calculated as described previously (32). The Bone Health Index (BHI) and its SDS (BHI-SDS) was calculated using the software BoneXpert from indices of three metacarpal bones as a parameter to approximate bone density from X-rays of the left hand (33).

### Development of Score and Expert Opinion

Definition: The terms “osteopathology” and “impaired bone health” are being used in this manuscript to describe different levels of skeletal late effects. For the purpose of this manuscript the term “osteopathology” refers to overt bone disease and the term “impaired bone health” refers to a condition with at least one pathological reading of the many assessed parameters of bone health. Bone health was assessed jointly by two clinical experts (C.G., B.H.). Both of whom are experienced pediatric endocrinologists. For information on the assessment process see below and Supplementary Material 1.

The core dataset for each patient comprised of 121 variables of which 56 were used for the expert assessment of the bone health status (Supplementary Material 1). The experts reviewed history, clinical and laboratory data of every patient to assign the bone health status into one of five categories: healthy, most likely healthy, osteopathology, most likely osteopathology, unable to determine. See Supplementary Material 2 for schematic representation of the stratification algorithm.

During the data analysis the challenge to define the status of bone health in individual patients due to a lack of a suitable scoring system became apparent. Therefore, for the purpose of this study, a subset of variables was used to assemble a score which we named Bone Pathology Harbinger—BPH. The variables were chosen based on the availability of age-specific reference ranges and/or evidence for osteopathology (e.g., pathological fracture). The following variables were included and scored if outside the normal range/pathological: (serum/plasma/urine) levels of (1) parathyroid hormone, (2) osteocalcin, (3) TSAP or BAP, (4) DPD, (5) urinary calcium to creatinine ratio, (6) pathological fractures after diagnosis of ALL, (7) knee pain on exercise or spontaneous back pain, and (8) bone mineral density reading (DXA Z < -2 in patients with normal height, or HAZ < -2 in patients with short stature). The sum of scored points divided by the number of possible points per patient represented the BPH score. The BPH therefore had a range from 0 (no pathology) to 1 (maximal pathology). It was calculated for 125 patients in whom at least 5 out of the 8 items were available.

### Statistics

Statistical analyses were performed using SAS 9.4 (SAS Institute, Cary, NC, USA) and PRISM for MAC 7.0 (GraphPad Software, Inc., La Jolla, CA, USA). Values are expressed as the mean +/- standard deviation (SD) and range unless stated otherwise. As in most of the variables normal distribution could not be assumed, associations between single variables were described by Spearman correlation coefficient. Differences in continuous variables between the groups were tested using the Mann-Whitney *U*-test for two-group comparisons, and the Kruskal-Wallis tests for more than two groups. Group differences in categorical variables were tested using the chi-square test statistics. The receiver operating curve (ROC) was calculated using the SAS macro as presented by Harris (34). For all tests, statistical significance was presumed at *P* < 0.05.

## Results

### Descriptive Statistics and Parameters of Bone Health

The mean age of the patients at follow up was 11.9 ± 4.76 years (2.6–20.9). Mean age at diagnosis was 6.07 ± 4.34 years (0.12–16.9), making for a mean of 5.88 ± 3.75 years after first diagnosis of ALL (range 0.59–13.9).

Eight patients of the cohort were in the second year of maintenance therapy. These patients were 1.33 years (±0.27) after initial diagnosis. The other 120 survivors were 4.59 years (±3.39 years) after the end of treatment (4.55 years ± 3.5 after end of chemotherapy; 4.72 ± 2.9 after BMT).

The mean height SDS was −0.37 ± 1.28 (-5.78–2.73), BMI SDS 0.37 ± 1.12 (-5.02–2.66). The Tanner stage SDS for pubic hair development and testicular volume/breast development was −0.16 ± 0.87 (−2.91–1.99) and −0.32 ± 1.0 (−3.39–1.79), respectively.

The questionnaire was completed by 120 of 128 survivors. Patients spent an average of 2.08 ± 1.41 h (0–5) of daily screen time. Of the patients three stated that they would spend most of the day non-ambulatory (sitting or lying down). Thirteen stated physical activity of up to three hours. One hundred four were physically active more than 3 h per day.

More detailed descriptive statistics of the 128 patients are displayed in Table 2. Of note, in Table 2 the male adult reference range is being displayed for the readers information. However, the applicable age-, sex-, and pubertal status adjusted reference ranges were used for the assessments of the individual data.

**Table 2:** Descriptive statistics of the cohort and parameters of bone health.

	**Mean**	**+/- SD**	**Range**	**Male adult normal range**	***N***
Age (years)	11.9	4.76	2.64–20.9		128
Age at diagnosis (years)	6.07	4.34	0.12–16.9		128
Peripheral blasts/μl at diagnosis	4077 (Median)	106810	0–597870		121
Time from initial diagnosis (years)	5.88	3.75	0.59–13.9		128
Height-SDS	−0.37	1.28	−5.78–2.73		127
BMI-SDS	0.37	1.12	−5.02–2.66		127
Delta bone age—biological age	−0.52	1.30	−4.35–3.09		59
PH-SDS	−0.16	0.87	−2.91–1.99		83
TV/BR-SDS	−0.32	1.00	−3.39–1.79		69
DXA Z	−0.38	1.18	−3.3–2.1		55
HAZ	0.13	1.06	−3.0–2.8		51
BHI-SDS	−0.86	1.12	−3.1–2.1		47
Serum-Calcium (mmol/l)	2.42	0.10	2.21–2.69		127
Serum-Phosphate (mmol/l)	1.40	0.24	0.68–2.42		127
25-OH V D (ng/ml)	16.3	10.6	1.85–62.6	> 10 (35)	124
1,25-(OH)_2_ V D (pg/ml)	60.9	24.3	13–158	35–90	116
TSAP (U/l)	211	98.9	37–468	25–124	127
BAP (U/l)	126	66.2	18.3–308	22–112	123
PTH (pg/ml)	46.0	25.8	9.60–160	15–65	122
NTX (nmolBCE/nmol crea)	408	281	21–922	34–780	31
DPD (μg/g crea)	147	86.4	27.3–363	110–450	69
Ca:Crea (mg/mg)	0.11	0.10	0.00–0.50	0.03–0.24 (36)	118
Osteocalcin (ng/ml)	103	63.7	11.9–426	18.6–71.5	79
IGF-1 SDS	−0.30	1.19	−3.55–2.93		128
Vitamin D intake (U/d)	93.6	66	1.2–277		118
Calcium intake (mg/d)	790	374	170–2247		118
Screen-time (h)	2.08	1.41	0–5		120

*Mean, SD, range, male adult normal values and the number of patients examined are displayed. body mass index (BMI) SDS, pubic hair stage (PH) SDS, testicular volume/breast development stage SDS (TV/BR) SDS, dual-energy X-ray absorptiometry (DXA) SDS, Height adjusted Z-scores (HAZ), Bone Health Index (BHI-SDS), 25-OH vitamin D (25-OH VD), serum 1,25-(OH)2 vitamin D (1,25-OH VD), total serum alkaline phosphatase (TSAP), bone-specific alkaline phosphatase (BAP), urinary N-terminal telopeptide (NTX), urinary deoxypyridinoline (DPD), urinary calcium to creatinine ratio (Ca:Crea), insulin-like growth factor-1 SDS (IGF-1 SDS), nutritional vitamin and calcium intake, and screen-time (h)*.

The frequency of aberrant biochemical and radiological findings on bone health are summarized in Table 3. In total in 77 % of patients any kind of bone health impairment was detected.

**Table 3:** Frequency of aberrant findings suggesting impaired bone health.

	**BPH score items**	**Fraction of patients affected**	**Percentage of patients affected**
Elevated PTH	•	18/122	14.8
Elevated or decreased TSAP/ BAP	•	43/128	33.6
Elevated or decreased DPD/NTX	•	10/98	10.2
Elevated or decreased Osteocalcin	•	11/79	13.9
Decreased urinary Ca excretion	•	3/118	2.5
Pathological fractures	•	8/121	6.6
Low BMD (DXA Z/HAZ < −2)	•	5/55	9.1
Bone pain (Knee or Back)	•	37/118	31.3
Osteonecrosis		16/128	12.5
Fractures of long bones		26/120 (4x2, 3x3)	15.9
25-OH VD deficiency (<20ng/ml)		89/124	71.8
Severe 25-OH VD def. (<10ng/ml)		43/124	34.7

*Number and percentage of patients affected by altered parameters of calcium metabolism, bone metabolism, or notable clinical findings are displayed. For the assessment of the calcium metabolism, the following parameters were used: decreased calcium excretion in the urine (Ca:Crea ratio <0.01 mg/mg). For the assessment of bone metabolism, the following parameters were used: elevated levels of urinary N-terminal telopeptide (NTX) or urinary deoxypyridinoline (DPD), elevated total serum alkaline phosphatase (TSAP) and/or bone-specific alkaline phosphatase (BSAP) and elevated PTH. Bone-related pain in form of knee pain associated with exercise and recurrent back pain in the last month as well as the history of fractures of long bones was also included. “•” indicates a variable which is included in the BPH score*.

### Bone Health According to Gender and Age

The mean DXA Z was significantly lower in female patients than in males (−1.06 vs. −0.08; SD 1.1 for both, *P* = 0.0035). This difference persisted when comparing the height adjusted Z-scores (HAZ, −0.44 ± 1.12 vs. 0.39 ± 0.94, *P* = 0.02). Osteonecrosis was present more often in female patients (12 vs. 4, *P* = 0.07). As expected, patients with osteonecrosis were older at diagnosis than patients without osteonecrosis 10.8 ± 4.39 years (3.25–16.9) vs. 5.39 ± 3.90 years (0.12–16.6). No further difference in bone health related parameters was detectable between male and female patients.

### Quantification of Bone Health via Expert Opinion and Bone Pathology Harbinger (BPH)

An expert opinion on the status of bone health was obtained for all patients. Thirty nine patients were labeled as healthy or most likely healthy and 23 patients as affected with osteopathologies or most likely affected with osteopathologies. In 63 patients a definite classification based on the available parameters by the expert was not possible. Of these, 46 patients were not categorizable due to unconcise or conflicting findings and data, 12 patients had osteonecroses but did not display any sign for osteopathologies otherwise. In five patients data was incomplete and therefore insufficient to assign them to any category.

**Figure 1:**
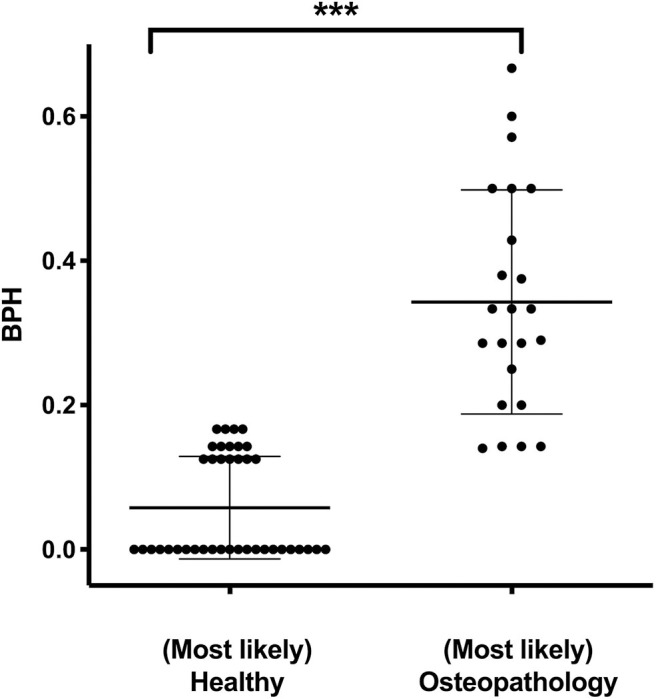
Expert opinion and the Bone Pathology Harbinger (BPH). BPH scores in patients who were assigned to the categories (most likely) healthy (*n* = 39) or (most likely) osteopathology (*n* = 23) by independent expert opinion. Lines indicate mean and standard deviation. Statistically significant differences between the groups, determined via Mann-Whitney test, are indicated with asterisks (***P < 0.001).

The median score of the BPH in the entire cohort was 0.14 (0–0.67; 25th percentile = 0, 75th percentile = 0.25; *n* = 125). In patients who were found to have (most likely) osteopathologies by the expert opinion, the BPH score was elevated compared to the patients labeled as (most likely) healthy (0.32 ± 0.17 vs. 0.06 ± 0.07; *P* < 0.001, Figure 1). To evaluate specificity and sensitivity of the BPH score for the study cohort, it was matched to the expert opinion. The resulting receiver operating curve features an area under the curve of 0.97. A cutoff of 0.16 for the BPH accounts for a sensitivity of 82 % and a specificity of 90 %.

### Bone Health and Calcium/Vitamin D Metabolism

**Figure 2:**
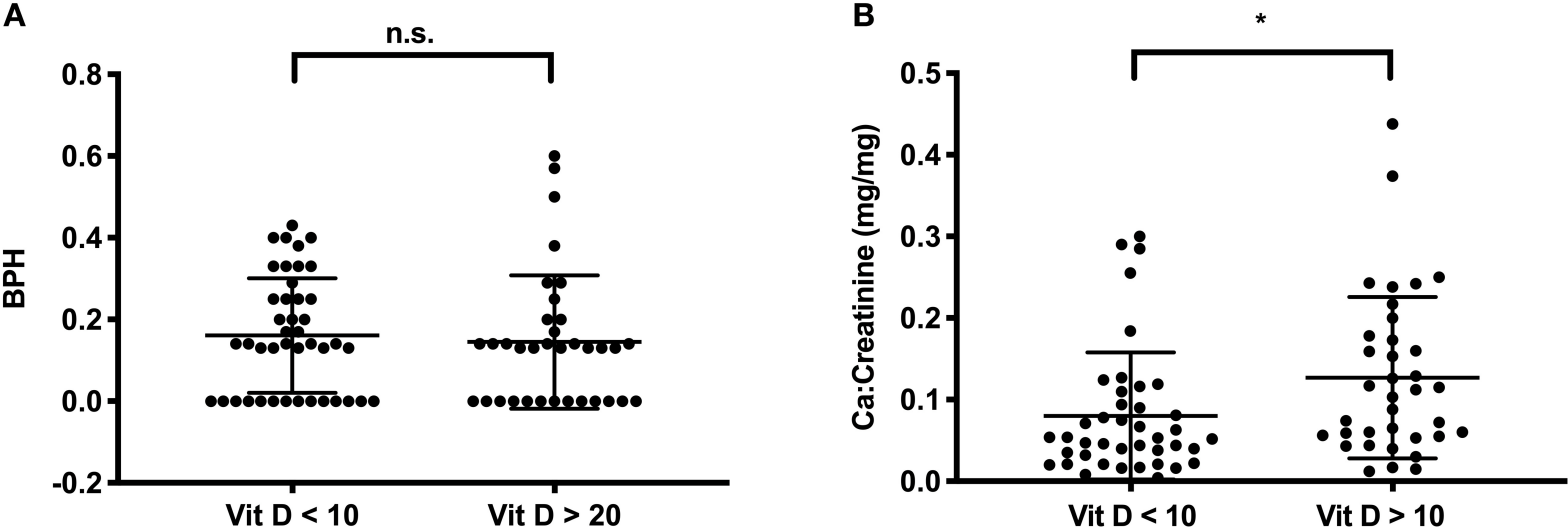
Vitamin D (in ng/ml) and calcium metabolism in survivors. **(A)** The BPH score is not different in survivors with severe Vitamin D deficiency (Vit D < 10 ng/ml, *n* = 43) or with sufficient vitamin D levels (Vit D > 20 ng/ml, *n* = 35); **(B)** Urinary Ca/Creatinine in the same group of patients. Lines indicate mean and standard deviation. Statistically significant differences between the groups, determined via Mann-Whitney test, are indicated with asterisks (*P < 0.05).

To evaluate the role of a 25-OH vitamin D deficiency for bone health in this cohort, we compared BPH scores in survivors with severe vitamin D deficiency [as defined by Holick, (35)], to those with sufficient levels. BPH scores did not differ between the groups (0.16 ± 0.14 vs. 0.17 ± 0.20, *P* = 0.35, Figure 2A). However, in the group of patients with sufficient vitamin D levels the calcium:creatinine ratios in urine were higher, indicating adequate calcium stores, compared to the group of patients who displayed severe vitamin D deficiency (0.13 ± 0.09 vs. 0.08 ± 0.08 mg/mg, *P* = 0.01, Figure 2B).

### Bone Health and Risk Groups/Treatments Groups and Age at Diagnosis

**Figure 3:**
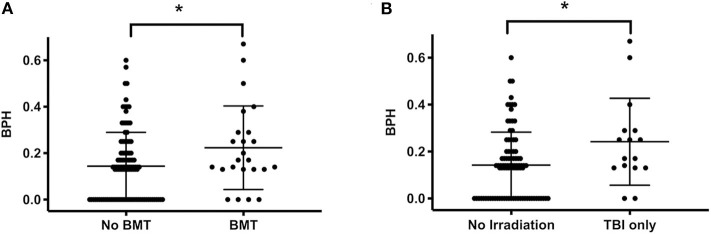
BPH score and bone marrow transplantation or total body irradiation. **(A)** The score in patients subjected to BMT (*n* = 24) is higher than in patients without BMT (*n* = 101, indicating impaired bone health following BMT). **(B)** Also, the score was higher in patients who underwent TBI (*n* = 16) vs. those without any irradiation (*n* = 92). Lines indicate mean and standard deviation. Statistically significant differences between the groups, determined via Mann-Whitney test, are indicated with asterisks (*P < 0.05).

Treatment stratification (standard and medium vs. high risk) did not affect the BPH score outcome (0.15 ± 0.15 vs. 0.19 ± 0.16, *P* = 0.25). However, the score was significantly higher in survivors after BMT compared to other survivors (0.22 ± 0.18 vs. 0.14 ± 0.15, *P* = 0.04, Figure 3A). This corresponds to a higher score in patients after total body irradiation compared with patients without irradiation (0.24 ± 0.19 vs. 0.14 ± 0.14, *P* = 0.03, Figure 3B).

The age at diagnosis differed between the groups labeled as (most likely) healthy vs. (most likely) osteopathologies. The healthy group being significantly younger at initial diagnosis (4.75 ± 3.36 vs. 7.91 ± 4.51 years, *P* = 0.001).

**Figure 4:**
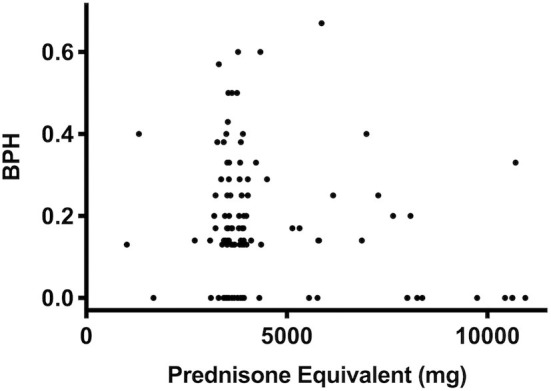
Prednisone equivalent dosage and BPH score as an indicator of bone health. In a bivariate regression the cumulative dose of prednisone equivalent did not correlate with score values (*P* = 0.4, *r* = 0.08, *n* = 123) in survivors of ALL.

Prednisone equivalent dosages varied markedly between patients, depending on intensity of treatment, occurrence of GvHD, etc. However, the cumulative dose of steroids did not correlate with score values (*r* = 0.08, *P* = 0.4, Figure 4). Also, the cumulative prednisone dose was not different between the group of patients labeled as affected by (most likely) osteopathology compared to the group labeled as (most likely) healthy (3,799 ± 2,384 mg vs. 3,829 ± 1,364 mg, *P* = 0.52). In addition, no correlation between bone health score and the dose of the putative bone damaging agent methotrexate (*r* = 0.01, *P* = 0.96) was found.

Focusing on fractures, the total prednisone equivalent dosage in patients with any fractures (*n* = 33) vs. no fractures did not differ (4,317 ± 1,708 mg vs. 4,487 ± 2,103 mg, *P* = 0.52), neither was a difference for pathological fractures (*n* = 8) vs. no pathological fractures detectable (4,606 ± 3,303 mg vs. 4,347 ± 1,720 mg, *P* = 0.31).

## Discussion

In juvenile survivors of ALL, a reduced bone mineral density and the occurrence of osteonecrosis have been previously described as indicators of impaired bone health (9, 37, 38). While the progression to overt osteopathologies may be preventable in many cases, sequelae from impaired bone health may contribute to increased morbidity and loss of quality of life in adulthood (as reviewed recently by 8). Therefore, efforts for detection and prevention of impaired bone health should be intensified in juvenile survivors. Identifying survivors at risk for impaired bone health requires not only the measurement of BMD but the evaluation of a spectrum of metabolic, hormonal, physical and lifestyle parameters. We aimed to describe the status of bone health thoroughly in this cross-sectional study in juvenile survivors of ALL.

To allow for a comprehensive assessment of bone health for the purpose of this study we used an expert opinion and compared it to a screening score (BPH). The score incorporates clinical, biochemical and radiographic parameters with defined reference values, which can easily be obtained in any follow-up setting. We found that the information of the score corresponded well to the evaluation of experienced experts, with a cutoff of 0.16 being highly specific and sensitively associated with impaired bone health. Such a score may become a helpful tool for pediatric oncologists to refer the patient to a pediatric bone specialist. Further modeling and validation in other cohorts of survivors and prospective long-term observation is therefore needed and well worth pursuing.

The hormone vitamin D and calcium as a nutritional component are known to be essential for bone metabolism (21, 39) and their deficiency is associated with impaired bone health in children and adults (40, 41). In this study, we detected a higher rate of severe vitamin D deficiency than expected given the known prevalence in healthy German children and adolescents (42). In this study, vitamin D deficiency was associated with a lower Ca/creatinine ratio and in 15% of survivors with (secondary) hyperparathyroidism, indicating a relevant negative calcium balance. Interestingly though, no difference in the BPH in patients with vitamin D deficiency or with vitamin D sufficient levels was observed. This is in line with the understanding that it is not the vitamin D deficiency itself, but the calcium deficiency, which is of relevance to bone health in children (39). Based on the results of this study, we have changed our routine clinical practice to a more frequent assessment of 25-OH vitamin D levels and a rigorous correction of Vitamin D deficiency in survivors of ALL.

However, in survivors of ALL, the finding of vitamin D deficiency and hyperparathyroidism is of importance in the context of the ongoing debate whether elevated PTH levels may increase the risk for relapse in hematological and non-hematological malignancies (43–45). One possible mechanism is a disruption of the bone marrow niche's homeostasis (46). Also, a recent study described lower overall survival of patients with suboptimal vitamin D levels after BMT (47). Although none of these findings prove causality, the vitamin D and PTH levels should be tightly controlled for in follow-up clinics and a routine vitamin D supplementation in survivors should be aimed for (48–50).

Fracture risk in this population was comparable to the report of te Winkel et al. who described a 17% 3 year risk in a population of 672 ALL survivors (51), but lower than that reported earlier by Nysom et al. who reported up to 55% fractures in a cohort over a median follow-up of 7.6 years after completion of therapy (52). DXA Z-scores were comparable to data, recently published by Inaba et al. (13). In general, studies reporting BMDs in closer proximity to the time of diagnosis describe lower BMDs (51). This indicates a time-dependent improvement of BMD after completion of oncological therapy (53). Lower Z-scores may also in part be explained by lower body height in the (CNS irradiated) population and could possibly be corrected by height-adapted re-calculation of the BMD [as discussed by Mäkitie et al. (15)]. The height- and age-adapted values for the Bone health Index (BHI-SDS) of BoneXpert derived from x-rays of the left hand (32) in this cross-sectional study do not indicate general impaired mineralization in the presented cohort. Nevertheless, a sufficient peak bone mass during and following pubertal development should be aimed for in all children with cancer (12).

The overall rate of osteonecroses in this cohort was not different from reported rates (54, 55). An increasing prevalence of osteonecroses with older age at diagnosis was observed in the present study like in others (56).

Frequent back pain and knee pain after exercise can be indicative of impaired bone health (57). About one third of the survivors in the present study reported recurrent bone pain in their questionaire.

Even though the intensity of therapy may vary markedly depending on the stratification into different risk groups, we could not attribute any differences in bone health status and BPH score on risk groups. In particular, it was surprising to see that neither the cumulative dose of prednisone nor the cumulative doses of methotrexate influenced the score values in this cohort. This is in contrast to the findings by Ward et al. (58) who showed that higher daily doses of prednisone proved a higher hazard for the development of vertebral fractures or non-vertebral fractures. However, the timing of these studies differs, since the present cohort exclusively consists of patients in follow-up clinic. It is likely that the vertebral fracture rate that is reported by Ward et al. as an early bone affection in ALL is present in the German population as well—however since no screening for vertebral fractures during intensive therapy is implemented, many of the lesser symptomatic fractures may not be reported, and may not be detectable at a later point in time due to healing/reshaping of the vertebrae. As expected, however, BMT as well as any irradiation was accompanied by higher BPH scores.

Limitations of this study are its cross-sectional design and the relatively young mean age of the cohort. With eight patients at the end of maintenance therapy, the cohort does not solely consist of survivors. Therefore, we are following up this group of survivors throughout puberty and through transition into adult follow up care. This will help to gain more insight into the development of the various outcome parameters of bone health with age.

The challenge to define the complex status of bone health led us to introduce the BPH score for the purpose of this study. In order to establish the score as a generalizable instrument for clinical use, however, it will have to be validated on at least one, best multiple, comparable data sets and preferably on a larger number of survivors. Parts of this larger dataset could be used as a training data set to improve the score. Here, a penalized regression model, like a Lasso model, could help selecting and weighing variables (59).

## Conclusion

In this cross-sectional study 18% of the adolescent survivors of ALL displayed overt osteopathologies by clinical expert assessment and 77% of the study population displayed evidence of impaired bone health in at least one of the investigated parameters. Major contributing factors are BMT, irradiation and older age of initial diagnosis. Vitamin D deficiency itself likely accounts for secondary hyperparathyroidism in some patients but was not associated with aggravated impairment otherwise.

Patients with ALL need thorough surveillance in follow up clinics to investigate bone health, since bone morbidity is common but still poorly understood. Vitamin D deficiency and secondary hyperparathyroidism are easy to detect and to avoid by supplementation. Early detection of impaired bone health and appropriate intervention may improve bone health in survivors. A bone health score may be a suitable tool for this endeavor. More research and prospective studies on this subject are much-needed.

## Data Availability Statement

All datasets generated for this study are included in the article/Supplementary Material.

## Ethics Statement

The studies involving human participants were reviewed and approved by Research Ethics Committee of the Medical Faculty, University of Duisburg-Essen # 12-4966-BO. Written informed consent to participate in this study was provided by the participants' legal guardian/next of kin and eligible patients.

## Author Contributions

CG and MS: study conception and design. PH, MS, CK, and MM: acquisition of data. BH, CG, and MS: analysis and interpretation of data. MS, CG, and PH: drafting and revising the manuscript. All authors contributed to the article and approved the submitted version.

## Conflict of Interest

The authors declare that the research was conducted in the absence of any commercial or financial relationships that could be construed as a potential conflict of interest.

## Abbreviations

25-OH VD: Serum levels of 25-OH vitamin D
1,25-(OH)_2_ VD: 1,25-(OH)_2_ vitamin D
BSAP: Bone-specific alkaline phosphatase
BMI: Body mass index
BMT: Allogeneic Bone Marrow Transplantation
BPH: Bone Pathology Harbinger
Ca:crea: Urinary calcium to creatinine ratio
DPD: Urinary deoxypyridinoline
FSH: Follicle-stimulating hormone
GvHD: Graft vs. Host Disease
IGF-1: Insulin-like growth factor-1
NTX: Urinary N-telopeptide
PTH: Parathyroid hormone
SD: Standard deviation
SDS: Standard deviation score
TSAP: Total serum alkaline phosphatase
TSH: Thyroid-stimulating hormone.

## References

[1] Pui C-H Yang JJ Hunger SP Pieters R Schrappe M Biondi A. Childhood acute lymphoblastic leukemia: progress through collaboration. J Clin Oncol. (2015) 33:2938–48. 10.1200/JCO.2014.59.1636 26304874 PMC4567699

[2] Hiroto I Greaves M Mullighan CG Acute lymphoblastic leukemia. Lancet. (2013) 381:62187–4. 10.1016/S0140-6736(12)62187-4 PMC3816716 23523389

[3] Pui C-H Evans WE Treatment of acute lymphoblastic leukemia. N Engl J Med. (2006) 354:166–78. 10.1056/NEJMra052603 16407512

[4] Brignardello E Felicetti F Castiglione A Chiabotto P Corrias A Fagioli F. Endocrine health conditions in adult survivors of childhood cancer: the need for specialized adult-focused follow-up clinics. Eur J Endocrinol. (2013) 168:465–72. 10.1530/EJE-12-1043 23258270

[5] Sklar CA Antal Z Chemaitilly W Cohen LE Follin C Meacham LR. Hypothalamic-Pituitary and growth disorders in survivors of childhood cancer: an endocrine society clinical practice guideline. J Clin Endocrinol Metab. (2018) 103:1–24. 10.1210/jc.2018-01175 29982476

[6] Jensen MV Rugbjerg K De Fine Licht S Johansen C Schmiegelow K Andersen KK Endocrine late effects in survivors of cancer in adolescence and young adulthood a Danish population-Based cohort study + invited commentary + supplemental content. JAMA Netw Open JAMA Netw Open. (2018) 11:180349–180349. 10.1001/jamanetworkopen.2018.0349 PMC6324403 30646084

[7] Barnes N Chemaitilly W. Endocrinopathies in survivors of childhood neoplasia. Front Pediatr. (2014) 2:1–12. 10.3389/fped.2014.00101 25295241 PMC4172013

[8] Marcucci G Beltrami G Tamburini A Body JJ Confavreux CB Hadji P. Bone health in childhood cancer: review of the literature and recommendations for the management of bone health in childhood cancer survivors. Ann Oncol. (2019) 30:908–20. 10.1093/annonc/mdz120 31111878

[9] Mostoufi-Moab S Halton J. Bone morbidity in childhood leukemia: epidemiology, mechanisms, diagnosis, and treatment. Curr Osteoporos Rep. (2014) 12:300–12. 10.1007/s11914-014-0222-3 24986711 PMC4131149

[10] Halton J Gaboury I Grant R Alos N Cummings EA Matzinger M. Advanced vertebral fracture among newly diagnosed children with acute lymphoblastic leukemia: results of the canadian steroid-associated osteoporosis in the pediatric population (STOPP) research program. J Bone Miner Res. (2009) 24:1326–34. 10.1359/jbmr.090202 19210218 PMC3890351

[11] Kawedia JD Kaste SC Pei D Panetta JC Cai X Cheng C. Pharmacokinetic, pharmacodynamic, and pharmacogenetic determinants of osteonecrosis in children with acute lymphoblastic leukemia. Blood. (2011) 117:2340–7. 10.1182/blood-2010-10-311969 21148812 PMC3062406

[12] van der Sluis I van den Heuvel-Eibrink M. Osteoporosis in children with cancer. Pediatr Blood Cancer. (2008) 50:474–8. 10.1002/pbc.21407 18064660

[13] Inaba H Cao X Han AQ Panetta JC Ness KK Metzger ML. Bone mineral density in children with acute lymphoblastic leukemia. Cancer. (2018) 124:1025–35. 10.1002/cncr.31184 29266176 PMC5821586

[14] Thomas IH Donohue JE Ness KK Dengel DR Baker KS Gurney JG. Bone mineral density in young adult survivors of acute lymphoblastic leukemia. Cancer. (2008) 113:3248–56. 10.1002/cncr.23912 18932250 PMC2597561

[15] Mäkitie O Heikkinen R Toiviainen-Salo S Henriksson M Puukko-Viertomies L-R Jahnukainen K. Long-term skeletal consequences of childhood acute lymphoblastic leukemia in adult males: a cohort study. Eur J Endocrinol. (2013) 168:281–8. 10.1530/EJE-12-0702 23197573

[16] Brennan BMD Mughal Z Roberts SA Ward K Shalet SM Eden TOB. Bone mineral density in childhood survivors of acute lymphoblastic leukemia treated without cranial irradiation. J Clin Endocrinol Metab. (2005) 90:689–94. 10.1210/jc.2004-1476 15562013

[17] Halton JM Atkinson SA Fraher L Webber C Gill GJ Dawson S Altered mineral metabolism and bone mass in children during treatment for acute lymphoblastic leukemia. J Bone Miner Res. (1996) 11:1774–83. 10.1002/jbmr.5650111122 8915786

[18] Argüelles B Barrios V Pozo J Muñoz MT Argente J. Modifications of growth velocity and the insulin-like growth factor system in children with acute lymphoblastic leukemia: a longitudinal study. J Clin Endocrinol Metab. (2000) 85:4087–92. 10.1210/jc.85.11.4087 11095437

[19] Davies JH Evans BAJ Jenney MEM Gregory JW. Skeletal morbidity in childhood acute lymphoblastic leukaemia. Clin Endocrinol (Oxf). (2005) 63:1–9. 10.1111/j.1365-2265.2005.02263.x 15963054

[20] Watsky MA Carbone LD An Q Cheng C Lovorn EA Hudson MM. Bone turnover in long-term survivors of childhood acute lymphoblastic leukemia. Pediatr Blood Cancer. (2014) 61:1451–6. 10.1002/pbc.25025 24648266 PMC4625912

[21] Saggese G Vierucci F Boot AM Czech-Kowalska J Weber G Camargo CA. Vitamin d in childhood and adolescence: an expert position statement. Eur J Pediatr. (2015) 174:565–76. 10.1007/s00431-015-2524-6 25833762

[22] Jackmann N Mäkitie O Harila-Saari A Gustafsson J Nezirevic Dernroth D Frisk P. Vitamin d status in children with leukemia, its predictors, and association with outcome. Pediatr Blood Cancer. (2020) 67:1–10. 10.1002/pbc.28163 31925904

[23] Möricke A Zimmermann M Valsecchi MG Stanulla M Biondi A Mann G. Dexamethasone vs prednisone in induction treatment of pediatric aLL: results of the randomized trial aIEOP-BFM aLL (2000). Blood. (2016) 127:2101–12. 10.1182/blood-2015-09-670729 26888258

[24] Schündeln MM Goretzki SC Hauffa PK Wieland R Bauer J Baeder L. Impairment of bone health in pediatric patients with hemolytic anemia. PLoS One. (2014) 9:e108400. 10.1371/journal.pone.0108400 25299063 PMC4191967

[25] van Buuren S Ooms J. Stage line diagram : an age-conditional reference diagram for tracking development. Stat Med. (2009) 28:1569–79. 10.1002/sim.3567 19260011

[26] Mul D Fredriks AM van Buuren S Oostdijk W Verloove-Vanhorick SP Wit JM. Pubertal development in the netherlands 1965-1997. Pediatr Res. (2001) 50:479–86. 10.1203/00006450-200110000-00010 11568291

[27] Greulich W Pyle S Radiographic Atlas of Skeletal Development of the Hand and Wrist. 2nd ed Stanford, CA: Stanford University Press (1959). 10.1097/00000441-195909000-00030

[28] Henzen C Therapie mit glukokortikoiden : risiken und nebenwirkungen. Schweizerisches Medizin-Forum. (2003) 19:442–6. 10.4414/smf.2003.04866

[29] Blum W Breier B. Radioimmunoassays for iGFs and iGFBPs. Growth Regul. (1994). 4 (Suppl).:11–9. 7515738

[30] Ellis KJ Shypailo RJ Hardin DS Perez MD Motil KJ Wong WW. Z score prediction model for assessment of bone mineral content in pediatric diseases. J Bone Miner Res. (2001) 16:1658–64. 10.1359/jbmr.2001.16.9.1658 11547835

[31] GE_Healthcare. Lunar enCORE Pediatrics Reference Data. Available online at: http://www3.gehealthcare.com/en/products/categories/bone_health/pediatrics. (2010).

[32] Schündeln MM Marschke L Bauer JJ Hauffa PK Schweiger B Führer-Sakel D. A piece of the puzzle: the bone health index of the boneXpert software reflects cortical bone mineral density in pediatric and adolescent patients. PLoS ONE. (2016) 11:e0151936. 10.1371/journal.pone.0151936 27014874 PMC4807844

[33] Thodberg HH van Rijn RR Tanaka T Martin DD Kreiborg S. A paediatric bone index derived by automated radiogrammetry. Osteoporos Int. (2010) 21:1391–400. 10.1007/s00198-009-1085-9 19937229 PMC2895878

[34] Harris K ROC hard? No, ROC made easy! Seattle, WA: SAS Glob Forum (2010) 1–12.

[35] Holick MF. High prevalence of vitamin d Inadequacy and implications for health - proQuest. Mayo Clin Proc. (2006) 81:353–73. 10.4065/81.3.353 16529140

[36] Matos V Melle G Van Boulat O Markert M Bachmann C Guignard J. Urinary phosphate/creatinine, calcium/creatinine, and magnesium/creatinine ratios in a healthy pediatric population. j pediatr. (1997) 131:252–7. 10.1016/S0022-3476(97)70162-8 9290612

[37] Mandel K Atkinson S Barr RD Pencharz P. Skeletal morbidity in childhood acute lymphoblastic leukemia. J Clin Oncol. (2004) 22:1215–21. 10.1200/JCO.2004.04.199 15051768

[38] Boot AM van den Heuvel-Eibrink MM Hählen K Krenning EP de Muinck Keizer-Schrama SM. Bone mineral density in children with acute lymphoblastic leukaemia. Eur J Cancer. (1999) 35:1693–7. 10.1016/S0959-8049(99)00143-4 10674015

[39] Gou GH Tseng FJ Wang SH Chen PJ Shyu JF Pan RY. Nutritional factors associated with femoral neck bone mineral density in children and adolescents. BMC Musculoskelet Disord. (2019) 20:1–10. 10.1186/s12891-019-2901-9 31699056 PMC6839089

[40] Greene DA Naughton GA. Calcium and vitamin-D supplementation on bone structural properties in peripubertal female identical twins: a randomised controlled trial. Osteoporos Int. (2011) 22:489–98. 10.1007/s00198-010-1317-z 20544178

[41] Kalkwarf HJ Khoury JC Lanphear BP. Milk intake during childhood and adolescence, adult bone density, and osteoporotic fractures in uS women 1 - 3. (2003). (July). 10.1093/ajcn/77.1.257 12499350

[42] Dortschy R Schaffrath Rosario A Scheidt-Nave C Thierfelder W Thamm M Gutsche J Bevölkerungsbezogene verteilungswerte ausgewählter laborparameter aus der studie zur gesundheit von kindern und jugendlichen in deutschland [KiGGS]. Beiträge zur Gesundheitsberichtserstattung des Bundes. (2009) 92–104. 10.25646/3144

[43] Radujkovic A Kordelas L Krzykalla J Beelen DW Benner A Lehners N. Pretransplant vitamin d Deficiency is associated with higher relapse rates in patients allografted for myeloid malignancies. J Clin Oncol. (2017) 35:JCO.2017.73.008. 10.1200/JCO.2017.73.0085 28771378

[44] Michels KB Xue F Brandt L Ekbom A. Hyperparathyroidism and subsequent incidence of breast cancer. Int J Cancer. (2004) 110:449–51. 10.1002/ijc.20155 15095313

[45] Li M Chen P Li J Chu R Xie D Wang H. Review: the impacts of circulating 25-Hydroxyvitamin d Levels on cancer patient outcomes: a Systematic review and meta-Analysis. J Clin Endocrinol Metab. (2014) 99:2327–36. 10.1210/jc.2013-4320 24780061

[46] Reagan MR Rosen CJ. Navigating the bone marrow niche: translational insights and cancer-driven dysfunction. Nat Rev Rheumatol. (2015) 12:154–68. 10.1038/nrrheum.2015.160 26607387 PMC4947935

[47] Beebe K Magee K McNulty A Stahlecker J Salzberg D Miller H. Vitamin d deficiency and outcomes in pediatric hematopoietic stem cell transplantation. Pediatr Blood Cancer. (2018) 65:2. 10.1002/pbc.26817 28960811

[48] Keum N Giovannucci E. Vitamin d supplements and cancer incidence and mortality: a meta-analysis. Br J Cancer. (2014) 111:976–80. 10.1038/bjc.2014.294 24918818 PMC4150260

[49] Chowdhury R Kunutsor S Vitezova A Oliver-Williams C Chowdhury S Kiefte-De-Jong JC. Vitamin d and risk of cause specific death: systematic review and meta-analysis of observational cohort and randomised intervention studies. BMJ. (2014) 348:1–13. 10.1136/bmj.g1903 24690623 PMC3972416

[50] Munns CF Shaw N Kiely M Specker BL Thacher TD Ozono K. Global consensus recommendations on prevention and management of nutritional rickets. J Clin Endocrinol Metab. (2016) 101:83–106. 10.1159/000443136 26745253 PMC4880117

[51] te Winkel ML Pieters R Hop WCJ Roos JC Bökkerink JPM Leeuw J a. Bone mineral density at diagnosis determines fracture rate in children with acute lymphoblastic leukemia treated according to the dCOG-ALL9 protocol. Bone. (2014) 59:223–8. 10.1016/j.bone.2013.11.017 24287213

[52] Nysom K Holm K Michaelsen KF Hertz H Müller J Mølgaard C. Bone mass after treatment for acute lymphoblastic leukemia in childhood. J Clin Oncol. (1998) 16:3752–60. 10.1200/JCO.1998.16.12.3752 9850018

[53] Mostoufi-Moab S Brodsky J Isaacoff EJ Tsampalieros A Ginsberg JP Zemel B. Longitudinal assessment of bone density and structure in childhood survivors of acute lymphoblastic leukemia without cranial radiation. J Clin Endocrinol Metab. (2012) 97:3584–92. 10.1210/jc.2012-2393 22865901 PMC3674298

[54] Kunstreich M Kummer S Laws H-J Borkhardt A Kuhlen M Osteonecrosis in children with acute lymphoblastic leukemia. Haematologica. (2016) 28:90–107. 10.3324/haematol.2016.147595 PMC5394877 27742768

[55] Kuhlen M Kunstreich M Krull K Meisel R Borkhardt A. Osteonecrosis in children and adolescents with acute lymphoblastic leukemia: a therapeutic challenge. Blood Adv. (2017) 1:981–94. 10.1182/bloodadvances.2017007286 29296741 PMC5737600

[56] Bürger B Beier R Zimmermann M Beck JD Reiter A Schrappe M. Osteonecrosis: a treatment related toxicity in childhood acute lymphoblastic leukemia (ALL)–experiences from trial aLL-BFM 95. Pediatr Blood Cancer. (2005) 44(September 2004):220–5. 10.1002/pbc.20244 15514916

[57] Neville KA Cohn RJ. Bone health in survivors of childhood cancer. Lancet Diabetes Endocrinol. (2015) 8587:10–1. 10.1016/S2213-8587(15)00029-7 25873573

[58] Ward LM Ma J Lang B Ho J Alos N Matzinger MA. Bone morbidity and recovery in children with acute lymphoblastic leukemia: results of a six-Year prospective cohort study. J Bone Miner Res. (2018) 33:1435–43. 10.1002/jbmr.3447 29786884

[59] Sauerbrei W Perperoglou A Schmid M Abrahamowicz M Becher H. State of the art in selection of variables and functional forms in multivariable analysis-outstanding issues. Diagnostic Progn Res. (2020) 4:1. 10.1186/s41512-020-00074-3 32266321 PMC7114804

