# Supplementary material for: Prevalence of Osteopathologies in Children and Adolescents After Diagnosis of Acute Lymphoblastic Leukemia
Source: Front Pediatr. 2020 Aug 26;8:509. doi: 10.3389/fped.2020.00509 (PMC7479062; doi:10.3389/fped.2020.00509)
Supplement: Supplementary file 2 [file Table_2.DOCX]

**Decision Making Guideline via Expert Opinion**

**Strata of Bone Health Status**

1. Healthy
2. Most likely healthy
3. Most likely osteopathology
4. Osteopathology
5. Not categorizable (unconcise/ conflicting findings)
6. Not categorizable (osteonecrosis only)

**Abbreviations**

- complete list of abbreviated variables see supplementary material 1
- LLN = lower limit of normal
- ONR = outside normal range
- WNL = within normal limits
- ULN = upper limit of normal

**Laboratory findings “Bone-Lab”**

- serum bone-specific alkaline phosphatase, BAP or total serum alkaline phosphatase, TSAP
- urinary deoxypyridinoline, DPD or urinary N-terminal telopeptide, NTX
- plasma parathyroid hormone PTH (pg/ml)
- serum osteocalcin, OC
- urinary calcium to creatinine ratio (Ca/Creat)

**Definitions**

1. Bone-Lab WNL= all laboratory findings “Bone-lab” within normal limits
2. bone pain = spontaneous back pain OR exercise related knee pain

**Stratum 1: healthy**

- Bone-Lab WNL

& serum calcium and phosphate WNL

& no pathological fractures

& no bone pain

& for ages > 12 years: breast/testicular volume SDS > -1.5 (or result missing)

& if DXA available, DXA-Z > - 0.5

OR if DXA available & height SDS > 2: do not use DXA results for assessment

**Stratum 2: most likely healthy**

- **same as 1 but BAP mildly elevated** (max. + **10%)**
- **same as 1 but DPD missing or mildly elevated/decreased** ± **10%**
- **same as 1 but puberty – SDS may be outside normal range**
- **same as 1 but Ca/Creat below LLN & normal vitamin D**
- **same as 1 but PTH mildly elevated** (max. + **10%)**
- **same as 1 but exercise related knee pain**

**Stratum 3: most likely osteopathology**

- 2 Bone-Labs ONR & bone pain
- 3 Bone-Labs ONR
- bone pain & DXA-Z < -1 (osteopenia)
- pathological fractures & 1 Bone-Lab ONR
- PTH > 100 pg/ml
- 2 Bone-Labs ONR & DXA < -2 (any height SDS)
- 2 Bone-Labs ONR & DXA-Z < -1 AND height SDS > 0
- 2 Bone-Labs ONR & vitamin D < 10 ng/ml if DXA < -1 (or not available)
- 2 Bone-Labs ONR & spontaneous back pain & Vitamin D < 20 ng/ml

**Stratum 4: osteopathology**

- DXA < -2 & height SDS < -1
- any vertebral fracture
- PTH elevated &AP/BAP elevated & Ca/Creat < 0.05 & DPD OR NTX ONR
- 3 Bone-Lab ONR & bone pain
- any 3 Bone-Lab ONR & Osteocalcin ONR
- 3 Bone-Lab ONR & osteonecrosis
- 3 Bone-Lab ONR & bone pain & osteopenia (DXA <-1)

**Stratum 5: not categorizable (unconcise/ conflicting findings)**

- underlying syndromic disease
- (25-OH-Vitamin D or PTH) & Ca/Krea & DPD/NTX missing & (no DXA or Height SDS < -3)
- questionnaire & two or more laboratory findings missing
- questionnaire & DXA & one or more laboratory findings missing

**Stratum 6: not categorizable, osteonecrosis**

- osteonecrosis only (no other detected clinical or laboratory finding)
